# A Rare Presentation of Gastric Plexiform Fibromyxoma: Diagnostic Challenges and Surgical Management

**DOI:** 10.7759/cureus.87318

**Published:** 2025-07-05

**Authors:** Danush Aditya Munikrishnan, Dinesh Mahalingam, Ramita Anand, Vathani Yudakar, M Varun Viswanathan

**Affiliations:** 1 Department of General Surgery, ESIC Medical College and Hospital, K.K. Nagar, Chennai, IND

**Keywords:** antropyloric region, exophytic growth, malena, subtotal gastrectomy, ulceroproliferative growth

## Abstract

A 59-year-old female presented to the hospital with acute abdominal pain. She reported a six-month history of melena, early satiety, postprandial fullness, unintentional weight loss, and loss of appetite. Contrast-enhanced CT of the abdomen revealed an exophytic mass in the antropyloric region, measuring approximately 4.2 cm. Ultrasonography showed a large ulceroproliferative lesion in the gastric antrum. The patient subsequently underwent a subtotal gastrectomy. The resected specimen measured 13 cm along the greater curvature and 7.5 cm along the lesser curvature. Gross examination of the serosal surface revealed a nodular area near the greater curvature. On cut section, a well-defined submucosal lesion measuring 3.5 × 3 × 1.9 cm with intact overlying mucosa was identified. The lesion appeared gray-white and firm and exhibited a characteristic whorled pattern, extending into the muscularis propria. A PET-CT scan revealed mild hepatomegaly with hepatic cysts, along with renal calculi in the right kidney. Histopathological examination of the lesion was followed by immunohistochemical analysis. The tumor cells tested negative for CD117, anaplastic lymphoma kinase, and pan-cytokeratin and positive for smooth muscle actin, vimentin, and desmin. The Ki-67 proliferation index was low, estimated at 10%. These findings were consistent with a diagnosis of plexiform fibromyxoma, a rare benign gastric mesenchymal tumor.

## Introduction

Plexiform fibromyxoma (PF) is a rare mesenchymal tumor that typically arises in the stomach and exhibits benign behavior [1]. It is often associated with the presence of the *MALAT1::GLI1 *fusion gene [1]. PF is among the rarest gastric mesenchymal tumors and was first described in 2007. To date, only 121 cases of gastric PF have been reported worldwide, with just two cases documented in India [2].

Due to its origin and imaging characteristics, PF can sometimes be misdiagnosed as gastrointestinal stromal tumor (GIST) or other types of gastric mesenchymal tumors. However, accurate diagnosis relies on recognizing its distinct morphology in combination with immunohistochemical and molecular features.

Histologically, PF is characterized by a plexiform arrangement of non-atypical spindle-shaped and ovoid cells within a myxoid stroma [2]. The cytoplasm typically shows strong eosinophilic staining, and the nuclei are round to oval. Mitotic figures are rare (fewer than 5 per 50 high-power fields), the Ki-67 proliferation index is very low (under 3%), and no cellular atypia is observed [2].

Immunohistochemically, the tumor cells are negative for CD117/c-KIT, discovered on GIST 1, CD34, desmin, and S100. In contrast, they show positive staining for smooth muscle actin (SMA), with weak positivity for caldesmon and succinate dehydrogenase complex iron sulfur subunit B [2].

## Case presentation

A 59-year-old female presented to the hospital with acute abdominal pain, along with a six-month history of melena, early satiety, postprandial fullness, unintentional weight loss, and loss of appetite. Abdominal CT imaging revealed a 4.2 cm exophytic mass in the antropyloric region, while ultrasonography identified a large ulceroproliferative growth in the antrum of the stomach. Consequently, she underwent a subtotal gastrectomy.

**Figure 1:**
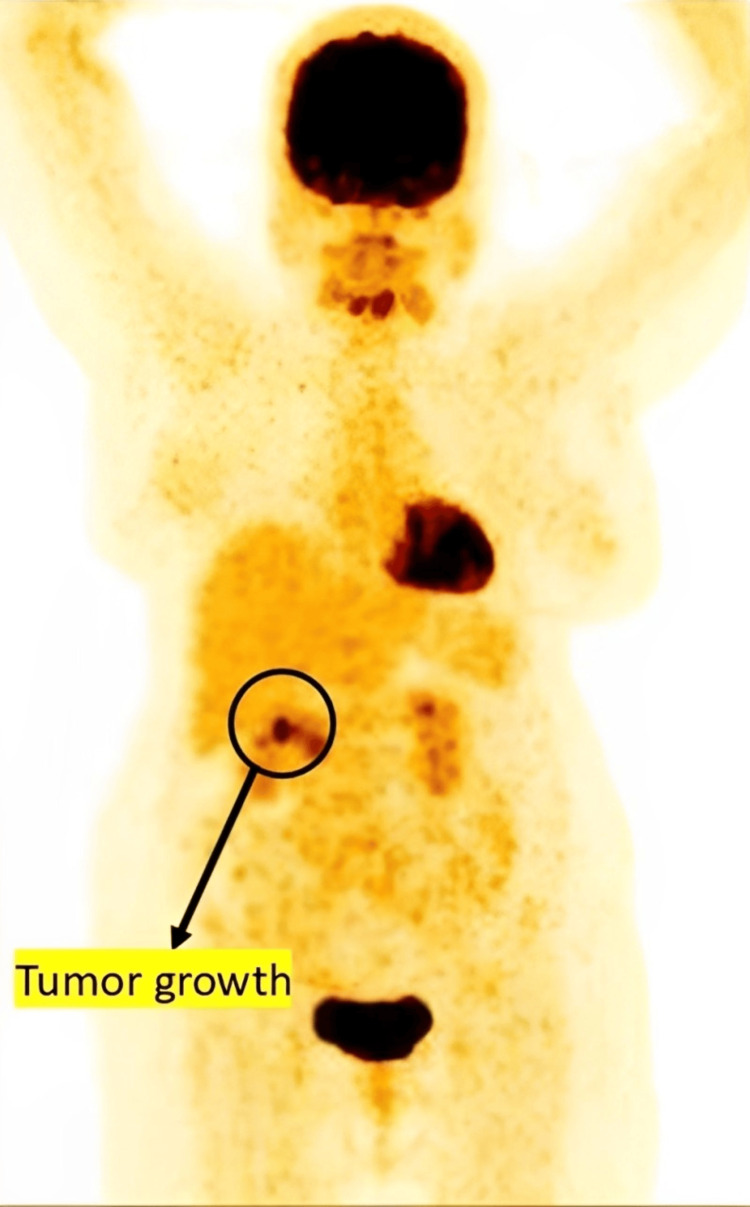
Preoperative PET-CT scan showing areas of increased metabolic activity

**Figure 2:**
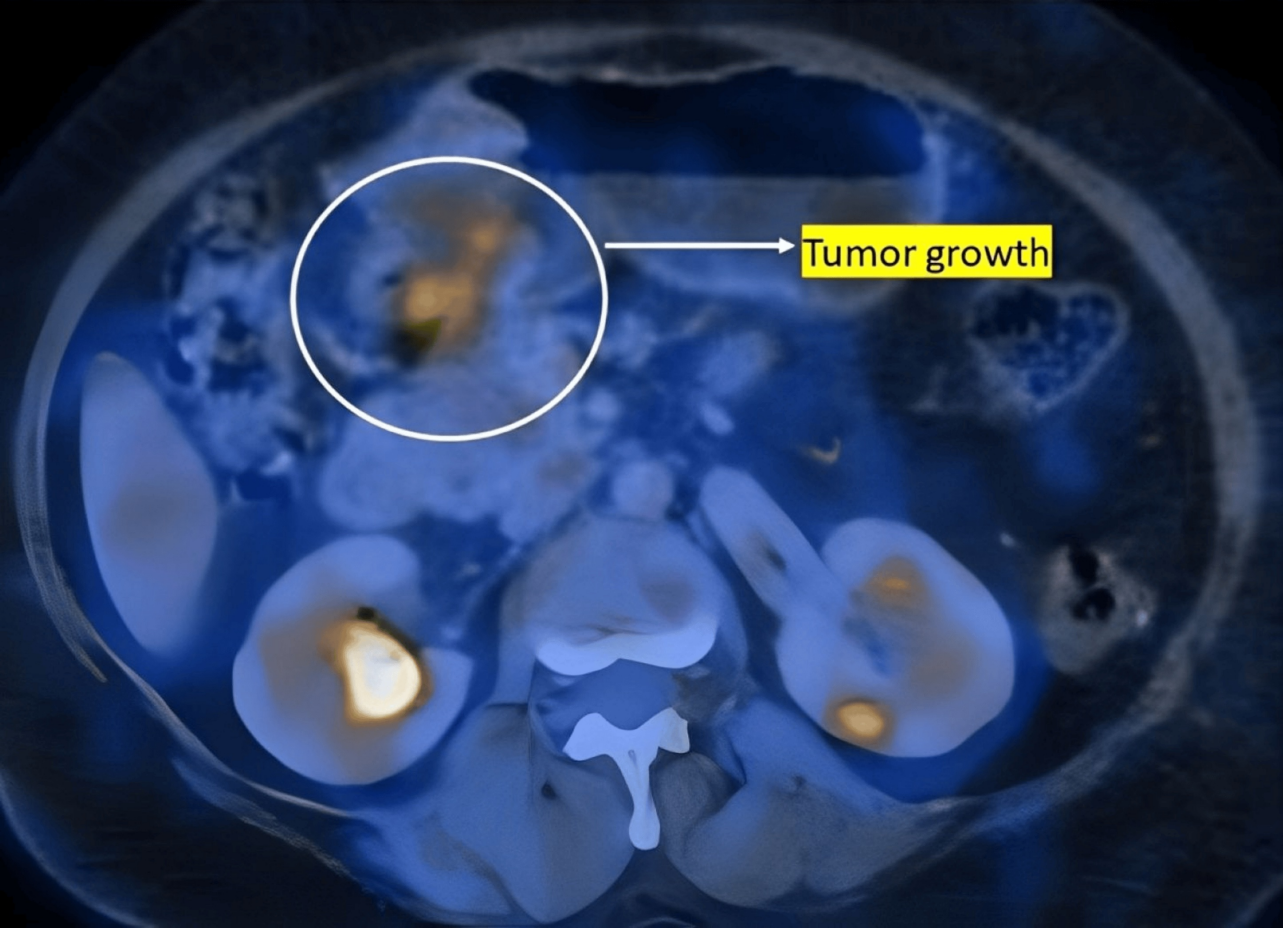
Axial PET-CT view demonstrating tumor growth

Figure 1 shows the preoperative PET-CT image, while Figure 2 shows the axial view. The fundus and body of the stomach appeared grossly distended, with a well-defined, non-homogeneously enhancing mass lesion involving the pyloric antral region along the greater curvature. Mild hepatomegaly with hepatic cysts was noted, along with right renal calculi. An irregular, heterogeneously enhancing soft tissue mass and proliferative, asymmetric mural thickening were observed in the anterolateral aspect of the antropyloric region, showing mild, patchy increased metabolic activity and resulting in partial luminal narrowing. Mild dilatation of the stomach proximal to the lesion was also seen. Two small nodular lesions closely abutting the primary mass, with suspicious loss of fat planes and minimal metabolic activity, were also identified. These PET-CT findings were suggestive of a primary gastric tumor with regional lymph node involvement.

A D2 distal gastrectomy with gastrojejunostomy was performed. The subtotal gastrectomy specimen measured 13 cm along the greater curvature and 7.5 cm along the lesser curvature. The serosal surface showed a nodular area near the greater curvature. On the cut section, the nodularity revealed a gray-white, firm lesion measuring 3.5 × 3 × 1.9 cm, with a whorled appearance and extension into the muscular layer of the stomach. The lesion was located 1.8 cm from the distal margin and 6.5 cm from the proximal margin. The adjacent gastric tissue appeared unremarkable.

A separately submitted omental specimen measured 27 × 20 × 1.5 cm. No solid areas were identified grossly. Serial sectioning of the omentum revealed two lymph nodes, measuring 1 × 0.7 × 0.3 cm and 0.9 × 0.8 × 0.3 cm, respectively. In addition, four gray-brown perigastric lymph node fragments were received together, measuring 1.5 × 1 × 0.5 cm.

**Figure 3:**
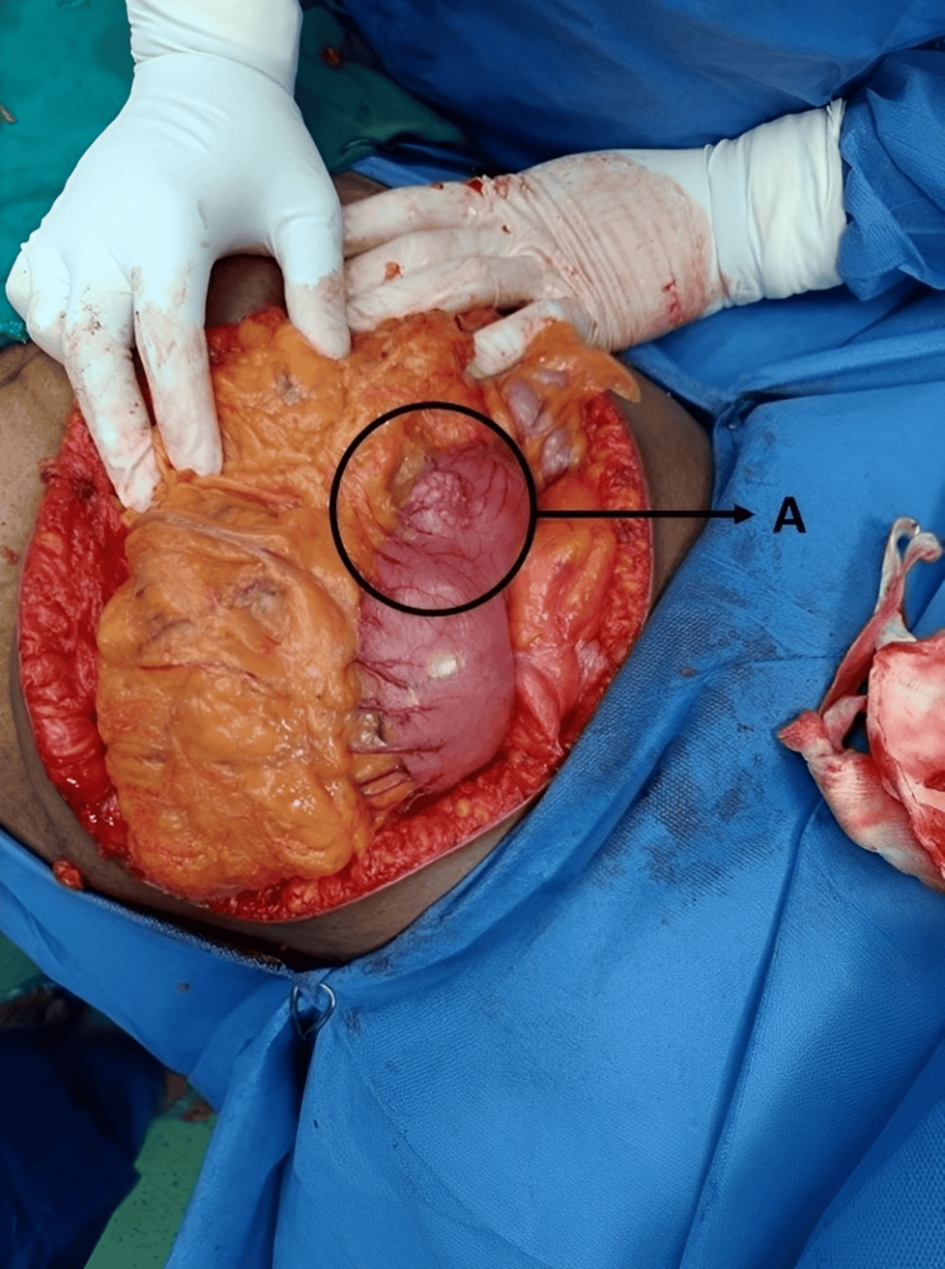
Intraoperative image showing nodular growth on the surface of the stomach Label A indicates the nodular growth pattern.

**Figure 4:**
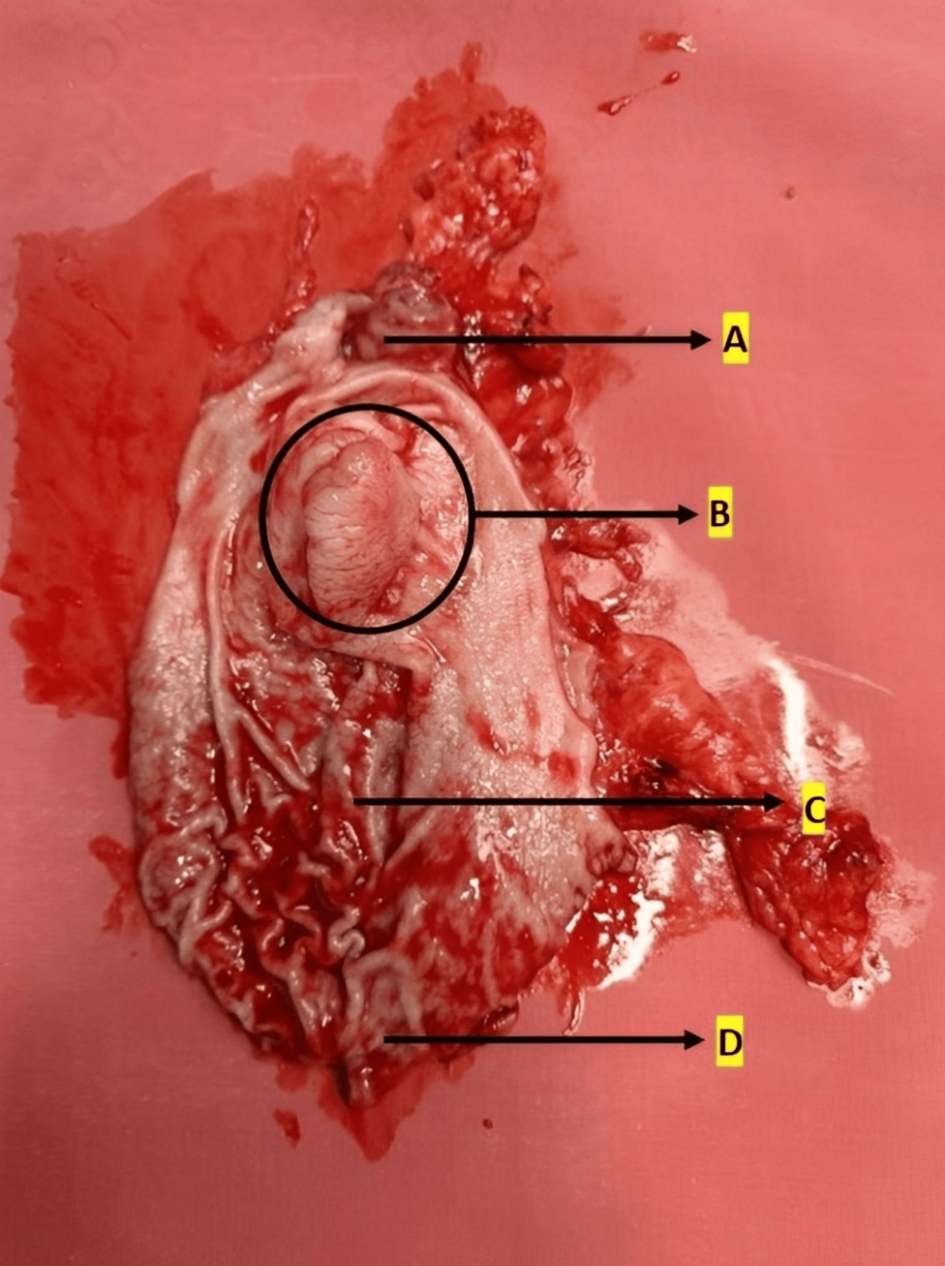
Labeled anatomical features of the resected specimen Label A indicates a portion of the lesser curvature of the stomach. Label B shows the exophytic tumor growth protruding into the gastric lumen. Label C highlights the gastric rugae. Label D indicates a portion of the greater curvature of the stomach.

**Figure 5:**
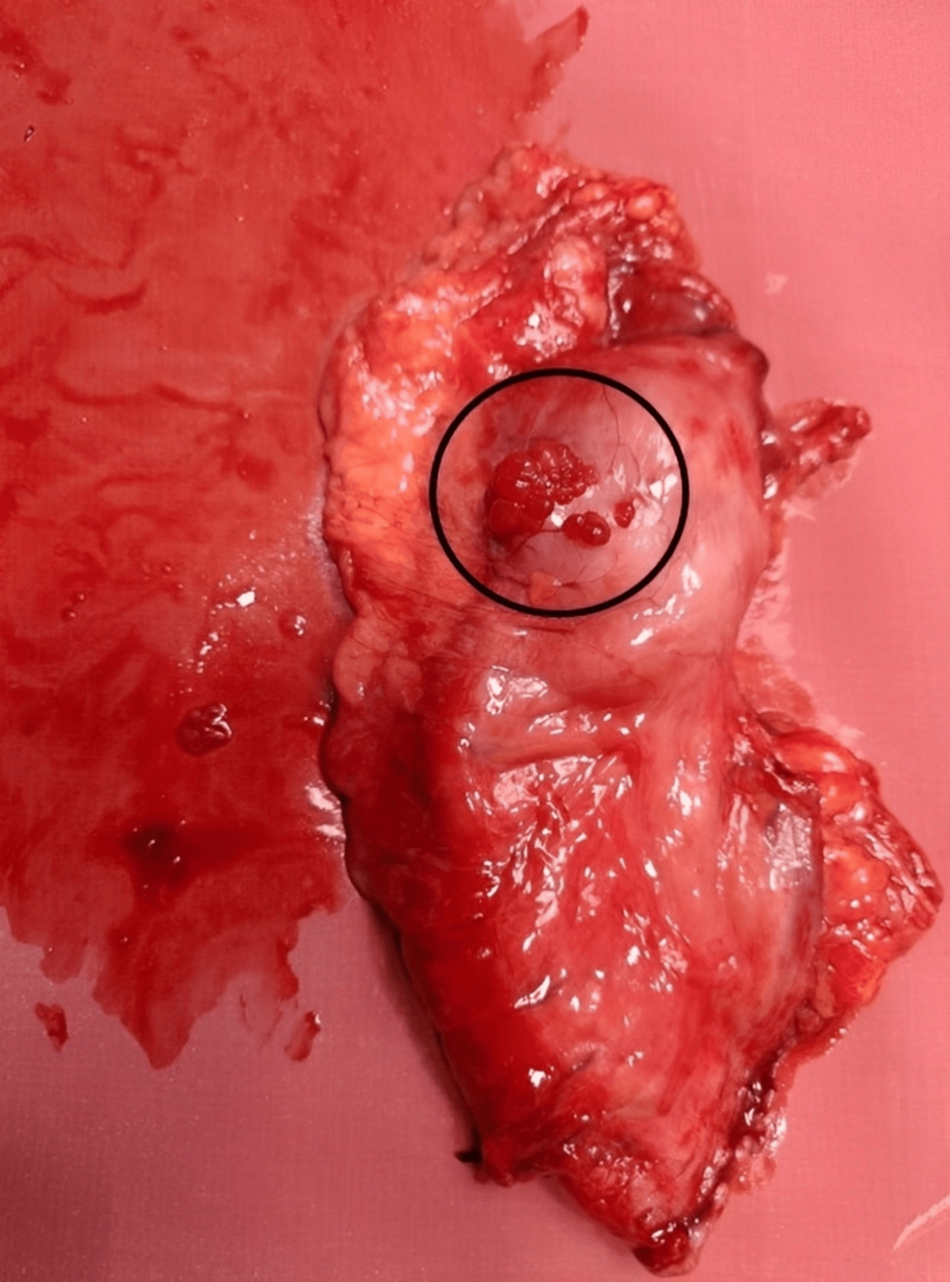
Gross image of the tumor growth

Figure 3 shows the nodular growth on the surface of the stomach, Figure 4 shows the labeled parts of the resected specimen, and Figure 5 shows the tumor growth.

Multiple sections showed a benign neoplasm composed of bland spindle cells embedded in a myxoid matrix with an arborizing, thin-walled capillary network. The mitotic rate was less than one mitosis per 10 high-power fields, and no cytological atypia was observed. Sections from the lymph nodes revealed two nodes with reactive lymphoid hyperplasia.

Immunohistochemical analysis demonstrated that the spindle-shaped cells were negative for CD117, anaplastic lymphoma kinase (ALK), and pan-cytokeratin, while showing positive reactivity for SMA, vimentin, and desmin. The Ki-67 proliferation index was low, at approximately 10%.

**Figure 6:**
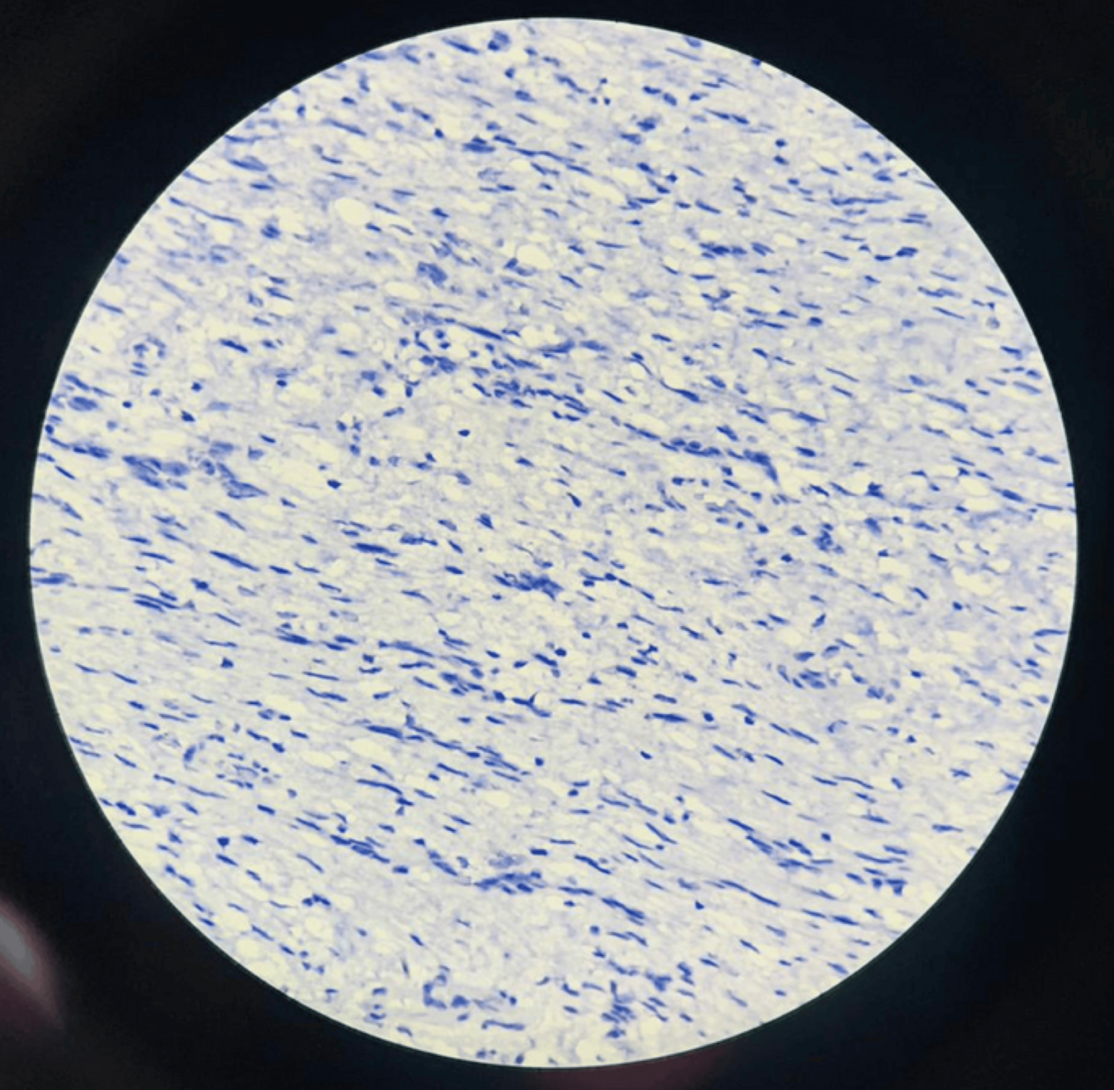
Immunohistochemical staining showing negative expression for CD117

**Figure 7:**
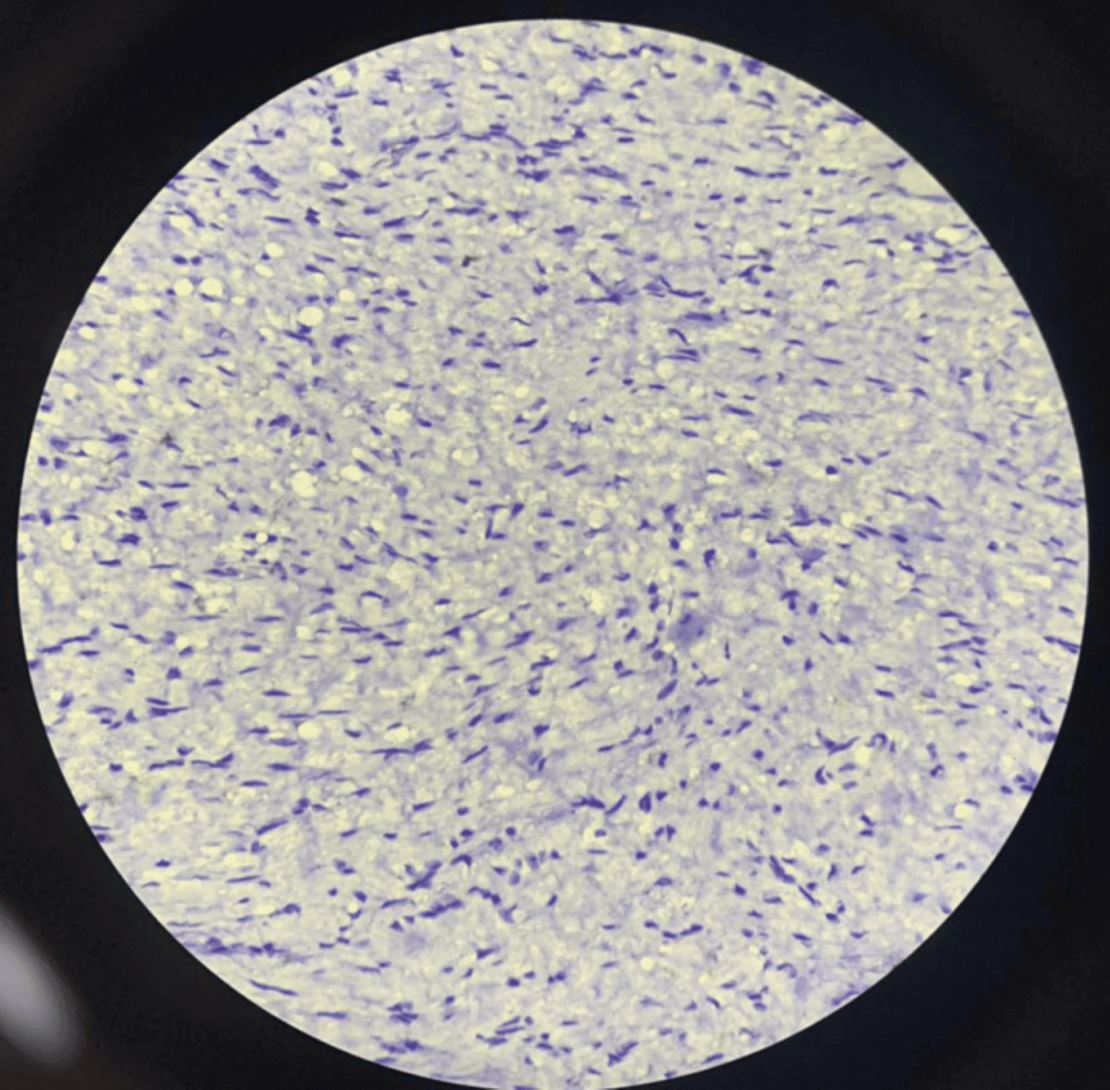
Immunohistochemical staining showing negative expression for ALK ALK, anaplastic lymphoma kinase

**Figure 8:**
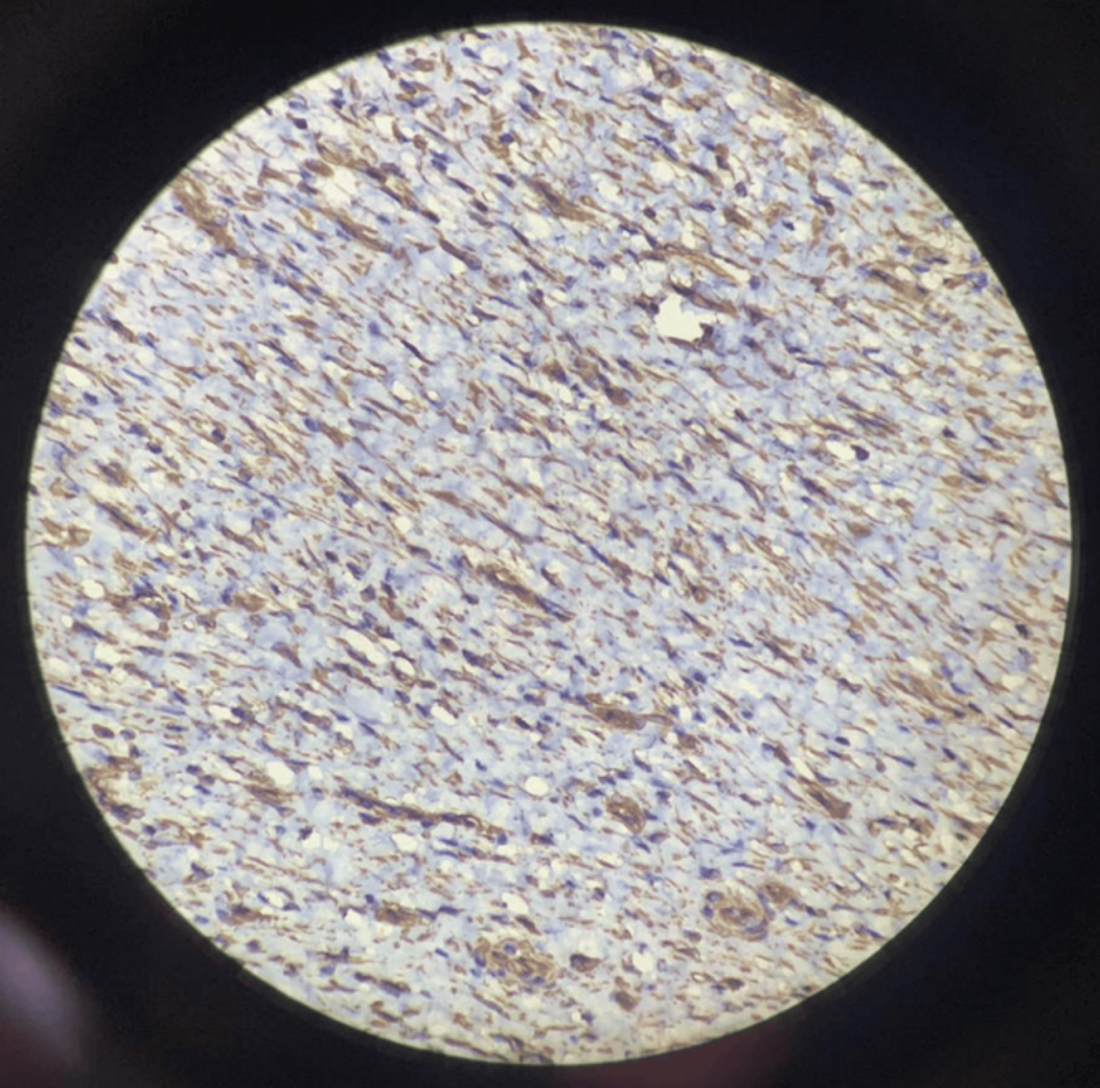
Immunohistochemical staining showing positive expression for SMA SMA, smooth muscle actin

**Figure 9:**
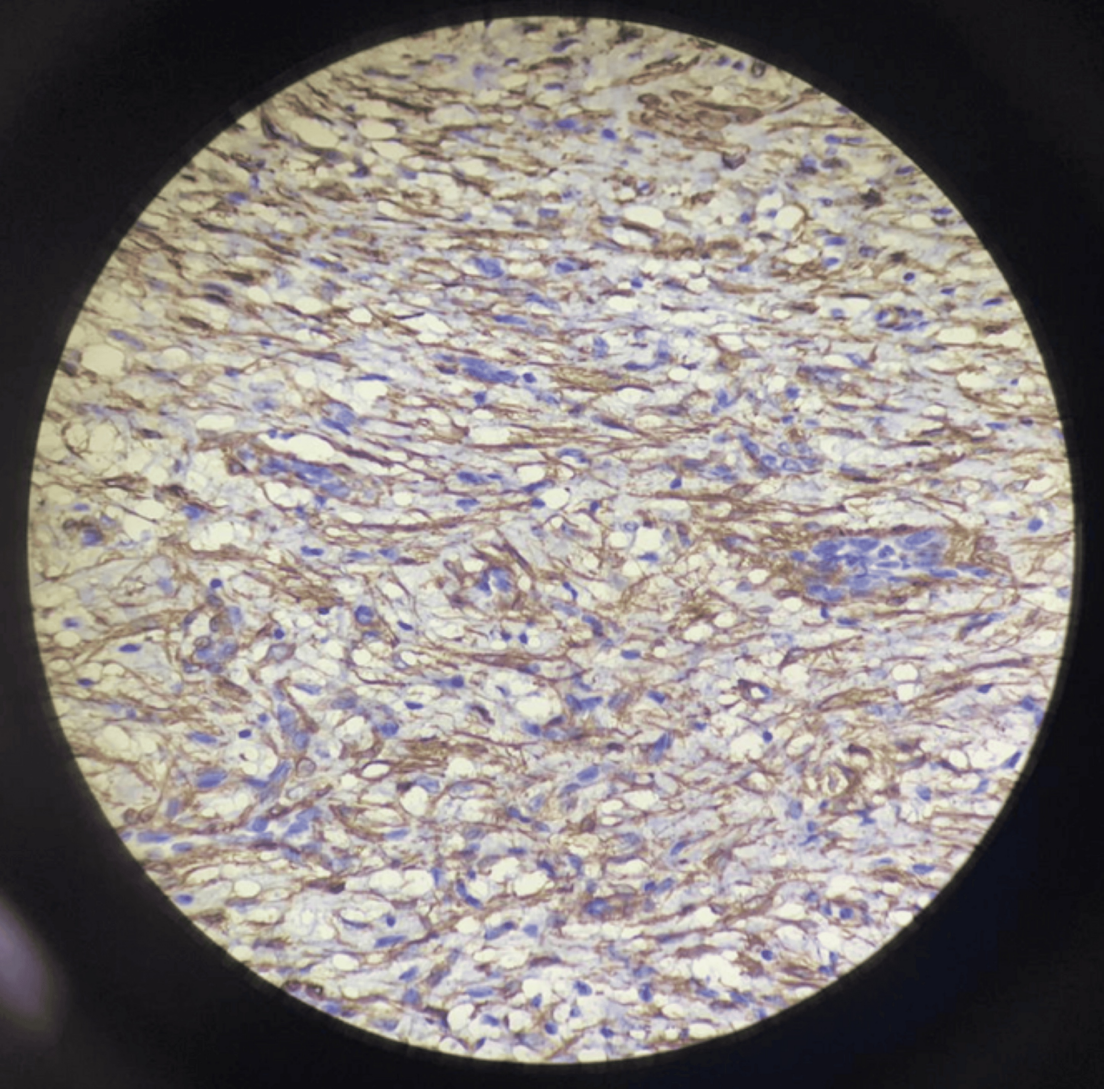
Immunohistochemical staining showing positive expression for vimentin

Figure 6 and Figure 7 show negative immunohistochemical staining for CD117 and ALK, respectively. Figure 8 and Figure 9 demonstrate positive staining of spindle-shaped cells for SMA and vimentin, respectively.

A total of six follow-ups were recommended for the patient. Of these, five were completed - four at three-month intervals and two at six-month intervals. During each follow-up, upper gastrointestinal endoscopy was performed, which revealed no lesions, with both the afferent and efferent loops appearing normal. Moving forward, the patient was advised to undergo annual upper gastrointestinal endoscopy. In the event of any recurrent lesions, further evaluation with a PET-CT scan will be conducted.

## Discussion

In 2007, Takahashi et al. published the first description of PF, also known as plexiform angiomyxoid myofibroblastic tumor (PAMT) [3]. PF often presents with nonspecific symptoms or signs of bleeding, which makes clinical diagnosis challenging [3]. A hallmark feature of PF, as reflected in its alternate name PAMT, is the presence of bland ovoid to spindle-shaped cells arranged in an irregular plexiform or multinodular pattern [3]. These cells are embedded in a myxoid and variably collagenized extracellular matrix, which is interspersed with a dense network of arborizing, capillary-sized blood vessels [3].

PF can occur across a broad age range, from seven to 75 years [4]. The main clinical symptoms include ulceration, upper gastrointestinal bleeding (hematemesis), and anemia. Additional symptoms may include nausea, vomiting, weight loss, the presence of a palpable gastric mass, and pyloric obstruction [4]. Key histological differential diagnoses include GIST, leiomyoma, leiomyosarcoma, schwannoma, desmoid fibromatosis, solitary fibrous tumor, inflammatory fibroid polyp, and inflammatory myofibroblastic tumor [4].

PF is a benign tumor characterized by hypervascularity, which contributes to its wide spectrum of clinical presentations. These range from incidental findings to nonspecific gastrointestinal symptoms, and in some cases, severe gastrointestinal hemorrhage. Common symptoms include vague abdominal pain, distension, and discomfort. Hemorrhagic manifestations are also frequent, often presenting as anemia, melena, or hematemesis [4-7].

In a study conducted by Zhou et al., histological examination revealed plexiform and multinodular involvement of the muscularis propria. The nodules varied in size and exhibited either well-defined borders or infiltrative margins that merged into adjacent normal tissue, occasionally coalescing into larger sheets. Within these nodules, bland spindle cells - with tapering ends, round to oval nuclei, fine chromatin, indistinct nucleoli, eosinophilic or amphophilic cytoplasm, and clearly defined cell borders - were dispersed throughout the matrix [6].

Although PF primarily affects the stomach, it can also occur in the small bowel [7]. In some cases, it may be entirely asymptomatic, as reported by Ebi et al. Diagnosing gastric PF preoperatively through imaging modalities such as gastrointestinal endoscopy, endoscopic ultrasound (EUS), CT, or MRI is typically difficult [8]. Furthermore, immunohistochemical analysis has limited diagnostic value, as PF non-specifically expresses markers like α-SMA, vimentin, and muscle-specific actin [8].

Comprehensive genomic profiling using next-generation sequencing has identified co-amplifications of *GLI1*, *CDK4*, and *MDM2*, along with mutations in the *TP53 *gene [1]. The World Health Organization officially adopted the term “plexiform fibromyxoma” in its 2010 classification of digestive system tumors to define this distinct entity [9].

## Conclusions

Diagnosing gastric PF preoperatively using imaging techniques, such as gastrointestinal endoscopy, EUS, CT, or MRI, is often challenging due to its nonspecific appearance. Despite its rarity, PF should be considered in the differential diagnosis of gastric subepithelial tumors, especially when imaging and endoscopic findings are inconclusive. Its clinical significance lies in its benign behavior, which distinguishes it from other gastric mesenchymal tumors that may require more aggressive management.

Accurate diagnosis relies on thorough histopathological evaluation, supported by immunohistochemical analysis, allowing for effective surgical treatment and the avoidance of unnecessary interventions. Clinical awareness of PF is crucial to guide appropriate management and ensure favorable patient outcomes. A detailed histomorphological assessment, together with targeted immunohistochemical testing, is essential to confirm the diagnosis and differentiate PF from other mesenchymal lesions. Although the pharmacological and molecular aspects of this tumor are not addressed in this report, future studies involving larger patient cohorts may help elucidate the underlying molecular mechanisms and identify novel diagnostic markers.
